# Assessment of Interobserver Reliability of Nephrologist Examination of Urine Sediment

**DOI:** 10.1001/jamanetworkopen.2020.13959

**Published:** 2020-08-21

**Authors:** Ragnar Palsson, Mia R. Colona, Melanie P. Hoenig, Andrew L. Lundquist, James E. Novak, Mark A. Perazella, Sushrut S. Waikar

**Affiliations:** 1 Renal Division, Brigham and Women’s Hospital, Boston, Massachusetts; 2 Division of Nephrology, National University Hospital of Iceland, Reykjavik, Iceland; 3 Renal Section, Department of Medicine, Boston University Medical Center, Boston, Massachusetts; 4 Division of Nephrology, Beth Israel Deaconess Medical Center, Boston, Massachusetts; 5 Division of Nephrology, Massachusetts General Hospital, Boston, Massachusetts; 6 Division of Nephrology, Henry Ford Hospital, Detroit, Michigan; 7 Section of Nephrology, Yale University School of Medicine, New Haven, Connecticut

## Abstract

**Importance:**

Urine sediment microscopy is commonly performed during the evaluation of kidney disease. Interobserver reliability of nephrologists’ urine sediment examination has not been well studied.

**Objective:**

Assess interobserver reliability of the urine sediment examination.

**Design, Setting, and Participants:**

In this diagnostic test study, urine samples were prospectively collected from a convenience sample of adult patients from an academic hospital in the United States undergoing kidney biopsy from July 11, 2018, to March 20, 2019. Digital images and videos of urine sediment findings were captured using a bright-field microscope. These images and videos along with urine dipstick results were incorporated in online surveys and sent to expert nephrologists at 15 US teaching hospitals. They were asked to identify individual sediment findings and the most likely underlying disease process.

**Exposures:**

Urine dipstick results and urine sediment images from patients undergoing native kidney biopsy.

**Main Outcomes and Measures:**

Interobserver reliability of urine sediment microscopy findings estimated by overall percent agreement and Fleiss κ coefficients. Secondary outcomes included concordance of diagnoses suspected by nephrologists with corresponding kidney biopsy results.

**Results:**

In total, 10 surveys from 10 patients containing 76 study questions on individual features were sent to 21 nephrologists, 14 (67%) of whom completed them all. Their combined 1064 responses were analyzed. Overall percent agreement for casts was an estimated 59% (95% CI, 50%-69%), κ = 0.52 (95% CI, 0.42-0.62). For other sediment findings, overall percent agreement was an estimated 69% (95% CI, 61%-77%), κ = 0.65 (95% CI, 0.56-0.73). The κ estimates ranged from 0.13 (95% CI, 0.10-0.17) for mixed cellular casts to 0.90 (95% CI, 0.87-0.94) for squamous epithelial cells.

**Conclusions and Relevance:**

In this study, substantial variability occurred in the interpretation of urine sediment findings, even among expert nephrologists. Educational or technological innovations may help improve the urine sediment as a diagnostic tool.

## Key Points

**Question:**

What is the interobserver reliability among practicing nephrologists when interpreting urine sediment findings?

**Findings:**

In this diagnostic study, 14 nephrologists provided 1064 interpretations of images of urine sediment findings. Agreement could be classified as slight, fair, moderate, substantial, or almost perfect. The interobserver reliability of urine sediment findings is mostly moderate to substantial but varies widely.

**Meaning:**

Results of this study suggest that efforts to decrease variability in urine sediment interpretations may help increase the yield of this widely used test in medicine.

## Teaser

This diagnostic study assesses interobserver reliability of nephrologist examination of urine sediment using high-resolution digital images and videos of the urine sediment of patients across the US undergoing kidney biopsy.

## Introduction

Microscopy of the urine sediment is a standard component of the complete urinalysis and among the oldest tests in medicine.^1^ Although in contemporary medical practice, urine microscopy is increasingly being performed in central laboratories by automated analyzers and technicians rather than clinicians, its interpretation continues to serve a role in the evaluation of patients with kidney disease.^2,3,4,5^ In spite of its long tradition, little is known about interobserver reliability of urine sediment examination among practicing nephrologists when interpreting urine sediment findings. Nephrologists often use their interpretation of a patient’s urine sediment to construct differential diagnoses and make decisions on whether to administer intravenous fluids, perform a kidney biopsy, initiate immunosuppressive therapy, or provide only supportive care. Given the commonly perceived importance of urine sediment examination in clinical decision-making, understanding variability in the urine sediment examination is important. However, a single study to our knowledge has been published on interobserver reliability of the nephrologist’s urine sediment examination.^6^ The primary aim of the present study was to examine interobserver reliability further by capturing high-resolution digital images and videos of the urine sediment of patients undergoing kidney biopsy and then obtaining independent interpretations of the imaged findings from nephrologists across the US. We secondarily explored how frequently nephrologists’ diagnostic impressions of urinalyses matched kidney biopsy results.

## Methods

### Microscopy and Imaging of Urine Sediment

We prospectively collected urine samples from a convenience sample of 10 adult patients (age ≥18 years) undergoing native kidney biopsy at Brigham and Women’s Hospital, Boston, Massachusetts, between July 11, 2018, and March 20, 2019. Within 2 hours after obtaining each urine sample, 10 mL were centrifuged at 1700 *g* for 5 minutes. The supernatant was discarded, and the sediment was resuspended and viewed unstained at low power (10 × objective) and high power (40 × objective) under a microscope (Nikon Eclipse 50i; Nikon Inc), which was set up for bright-field microscopy and allowed for polarization. Several still photographs (~5.9 megapixels; Nikon DS-Fi3; Nikon Inc) were obtained of each sediment. Along with each photograph, we captured a 10- to 15-second video showing the same field of view while shifting the focus plane up and down through the visualized sediment findings. Longer videos, approximately 1 minute each, were also obtained both at low power and high power while scanning across the microscopy slide to capture the overall appearance of the sediment. Results of urine dipstick tests were simultaneously recorded, as were the results of the nearest urinalysis reported from our hospital’s central laboratory which uses automated analyzers (Iris iQ200; Beckman Coulter Inc). Kidney biopsy results were obtained from patient medical records. The study was performed in accordance with the principles of the Declaration of Helsinki^7^ and approved by the Partners Human Research Committee, which granted a waiver of informed patient consent owing to the nature of this study. This study followed the Standards for Reporting of Diagnostic Accuracy (STARD) reporting guideline where applicable for diagnostic studies.

### Generation of Surveys and Their Review by Nephrologists

**Figure 1.**
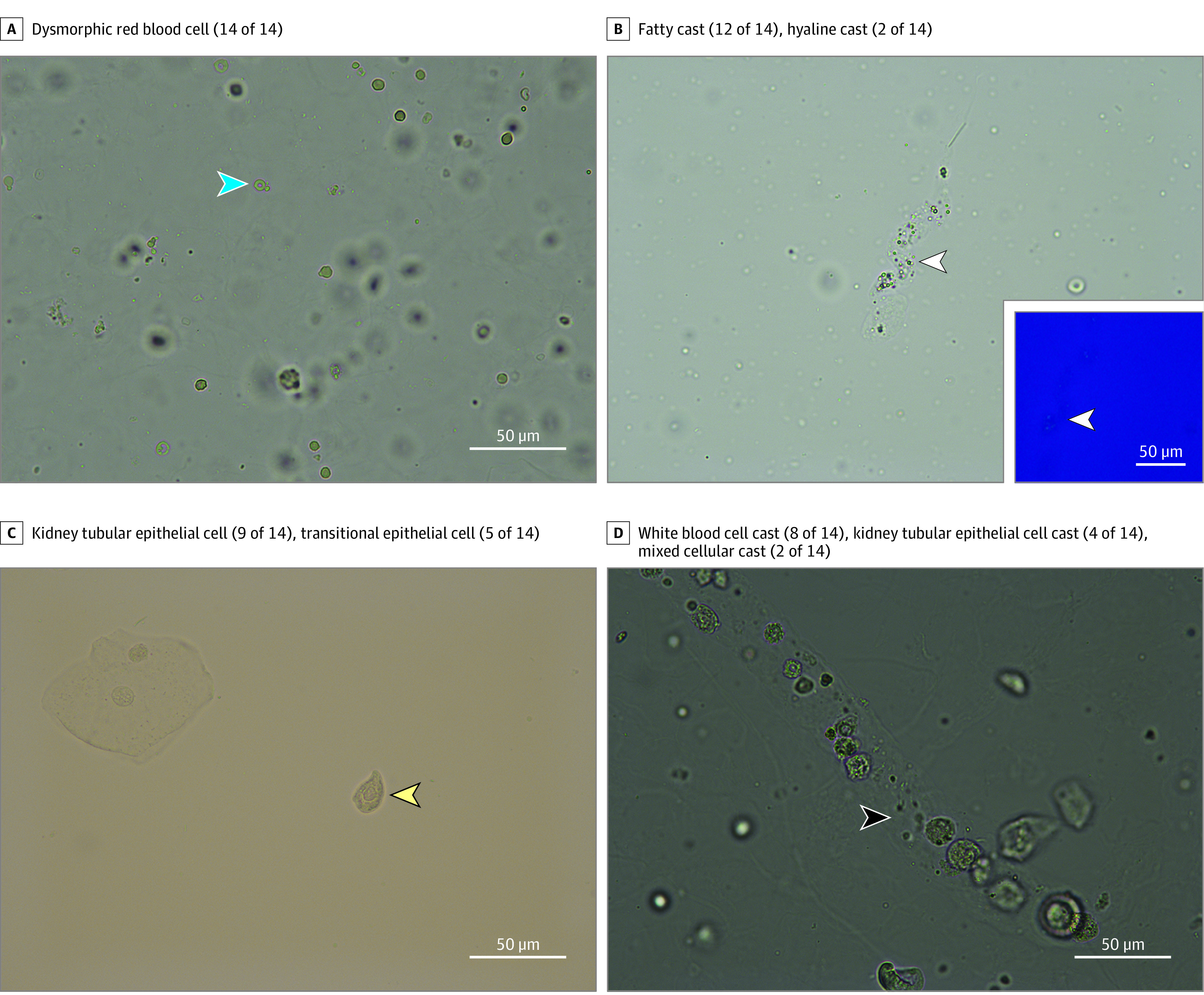
Example Digital Images Reviewed by Nephrologists Images were obtained at high power (40 × objective). Arrowheads indicate findings of interest that nephrologists were asked to identify. Their responses (No.) are shown above each image. The inset of panel B is the same field under polarized light.

For each patient, we created a deidentified online survey, showing first the urine dipstick results, then several still photographs with corresponding videos of individual urine sediment findings, and then the longer overview videos. The surveys were sent to 21 nephrologists at 15 academic hospitals across the US who had agreed to offer independent interpretations of the visualized sediment findings. The participating nephrologist reviewers had either been contacted directly by 2 of us (R.P. and S.S.W.) based on known clinical expertise or been referred to us by those to whom we had reached out. In each survey, the reviewers were asked to first identify the individual findings of interest, which had been marked by arrowheads in the still photographs (Figure 1). After reviewing the dipstick results and all available images and videos, the reviewers were asked to identify the underlying disease process without receiving further clinical information. An example of one of the surveys is available in the eAppendix in the Supplement. In addition, the reviewers were asked to complete a 1-time questionnaire about their views on the urine sediment examination as a diagnostic test and their use of it in practice.

### Statistical Analysis

We examined interobserver reliability separately for casts and other elements of the urine sediment. We did not analyze interobserver reliability of crystals because of the small number of crystals present. For descriptive purposes, in the absence of a reference standard, we initially interpreted the most frequent response to each question as the correct answer and used these designations to describe the mean percent agreement for each specific type of cast or sediment particle. Overall percent agreement for casts and other sediment elements was also calculated and required no specification of correct responses. To account for chance agreement and avoid the need to designate correct responses, we then estimated the Fleiss κ for casts (grouped into 8 categories) and other elements (grouped into 11 categories). To allow for these calculations, only the responses of reviewers who responded to all questions were included in the analysis. We estimated that 10 cases would provide sufficient variability in sediment findings; formal power calculations were not performed. We interpreted agreement as slight for κ = 0.00-0.20, fair for κ = 0.21-0.40, moderate for κ = 0.41-0.60, substantial for κ = 0.61-0.80, and almost perfect for κ > 0.80.^8^ The exploratory analysis of the concordance of diagnoses identified by nephrologists with kidney biopsy results was summarized descriptively. Because there is no agreed upon reference standard for the interpretation of the urine sediment, we did not compare results against a reference test and therefore do not report sensitivity, specificity, or positive or negative predictive values. Calculations were performed in Microsoft Excel v1905 (Microsoft Corporation) and Stata 14.2 (StataCorp LLC).

## Results

We sent reviewers 10 surveys, each containing deidentified images and videos from an individual biopsied patient. Together, the surveys contained images and videos asking for the identification of 37 casts and 39 other features such as cells, lipid, bacteria, or artifacts. A total of 14 reviewers (67%) answered every question and their combined 1064 responses to these questions on specific findings were included in the analysis of interobserver reliability. All 14 reviewers, after additionally reviewing the urine dipstick data and the scanning videos of the urine sediment at low power and high power, also determined what they believed to be the most likely diagnosis in every case.

**Figure 2.**
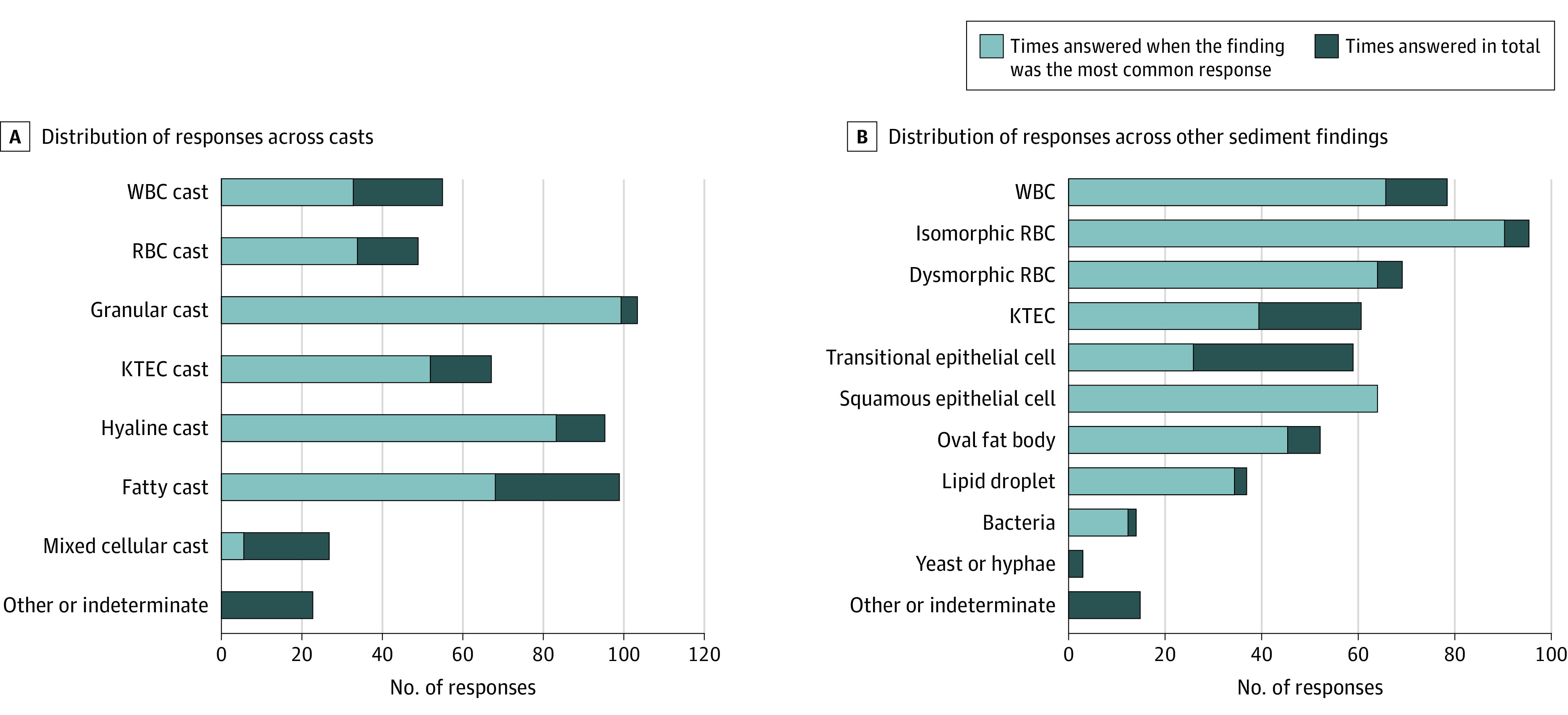
Distribution of Responses For each type of response listed, the number of times that it was chosen by the reviewers while representing the most common answer to a given image is shown in gray. In total, the 14 reviewers provided 1064 responses to the questions asking them to identify individual sediment findings included in this analysis. KTEC indicates kidney tubular epithelial cell; RBC, red blood cell; and WBC, white blood cell.

Figure 2 shows the distribution of responses made by the reviewers when they were asked to identify individual sediment findings marked into the survey images. Table 1 shows the number of pictures of different types of casts and other sediment findings that were sent to the reviewers, as determined by the most common response to each picture. The mean percent agreement for each type of sediment finding, along with the κ statistics, are presented in Table 1.

**Table 1.** Interobserver Agreement of Casts and Other Sediment Findings

Finding	No.^a^	Mean agreement, %	κ (95% CI)
Cast type^b^			
Hyaline cast	7	81.6	0.75 (0.71-0.78)
Granular or muddy brown cast	9	78.6	0.74 (0.71-0.78)
Fatty cast	6	80.9	0.53 (0.50-0.57)
Kidney tubular epithelial cell cast	6	61.9	0.49 (0.46-0.53)
Red blood cell cast	4	60.7	0.38 (0.35-0.41)
White blood cell cast	4	58.9	0.35 (0.31-0.38)
Mixed cellular cast	1	42.9	0.13 (0.10-0.17)
Other sediment findings^b^			
Squamous epithelial cell	5	91.4	0.90 (0.87-0.94)
Isomorphic red blood cell	7	94.0	0.85 (0.81-0.88)
Dysmorphic red blood cell	5	91.4	0.83 (0.80-0.86)
Bacteria	1	85.7	0.72 (0.69-0.75)
Lipid droplet	3	81.0	0.72 (0.68-0.75)
White blood cell	6	77.4	0.62 (0.58-0.65)
Oval fat body	5	64.3	0.58 (0.55-0.62)
Transitional epithelial cell	2	92.9	0.48 (0.45-0.52)
Kidney tubular epithelial cell	5	55.7	0.29 (0.26-0.33)

^a^ No. of pictures of different types of casts and other sediment findings as determined by the most common response, which was used to calculate mean percent agreement.

^b^ Shown are cast types which on at least 1 occasion represented the most common response.

For casts, the estimated overall percent agreement was 59% (95% CI, 50%-69%) and the overall κ was 0.52 (95% CI, 0.42-0.62). The highest interobserver reliability as measured by κ was found for hyaline casts (0.75; 95% CI, 0.71-0.78) and granular or muddy brown casts (0.74; 95% CI, 0.1-0.78). Interobserver reliability was slight for mixed cellular casts (κ = 0.13; 95% CI, 0.10-0.17) and fair for white blood cell (WBC) casts (κ = 0.35, 95% CI, 0.31-0.38).

For particles in the urine sediment other than casts, overall percent agreement was 69% (95% CI, 61%-77%) and the overall κ was 0.65 (95% CI, 0.56-0.73). Interobserver reliability was highest for squamous epithelial cells (κ = 0.90; 95% CI, 0.87-0.94), isomorphic red blood cells (RBCs) (κ = 0.85; 95% CI, 0.81-0.88), and dysmorphic RBCs (κ = 0.83; 95% CI, 0.80-0.86). The lowest κ statistics were seen for kidney tubular epithelial cells (κ = 0.29; 95% CI, 0.26-0.33) and transitional epithelial cells (κ = 0.48; 95% CI, 0.45-0.52).

**Figure 3.**
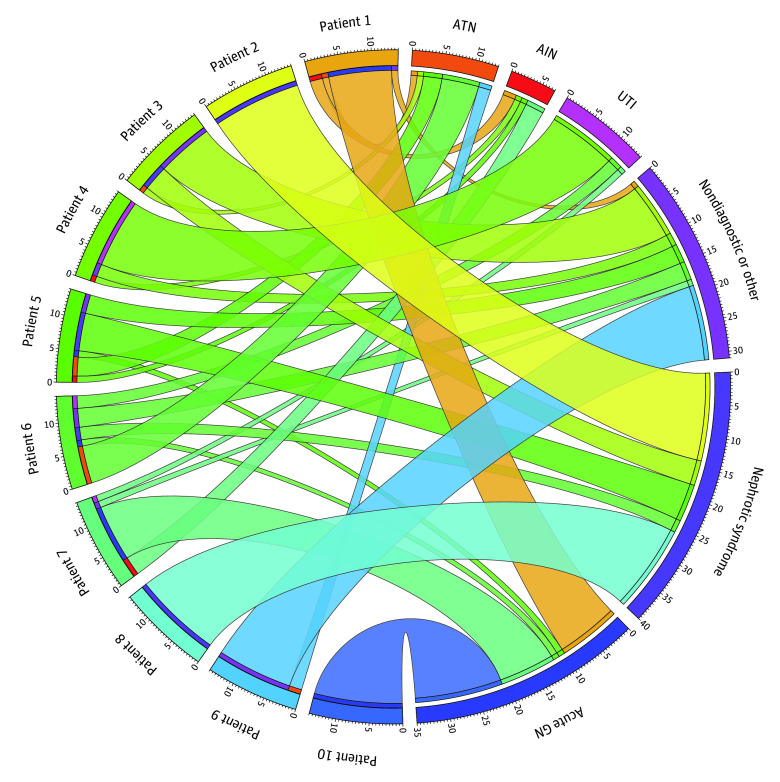
Chord Diagram Depicting Disease Process Suspected Based on Urinalysis Findings The chord diagram depicts the underlying disease process suspected by 14 nephrologists after their review of urinalysis data and urine sediment images from 10 patients undergoing kidney biopsy. Individual cases listed from 1 to 10 on the left side of the diagram correspond to the listing in Table 2, in which the clinicopathologic diagnoses made after kidney biopsy are presented. The width of each chord is determined by the number of nephrologists who gave the same answer. The total number of times each diagnostic category was chosen during the course of the study is also shown next to the segments representing the individual categories on the right side of the figure. AIN indicates acute interstitial nephritis; ATN, acute tubular necrosis; GN, glomerulonephritis; and UTI, urinary tract infection.

The disease processes believed most likely to be present in each case based on the reviewers’ evaluation of the urine sediment findings in comparison with the diagnoses made after kidney biopsy are presented in Table 2 and depicted in Figure 3. Agreement varied considerably between cases but was highest when glomerular pathology was present. In 3 cases, all 14 reviewers suspected the same underlying disease process with perfect agreement, which was, in turn, consistent with the findings on biopsy. Results of urinalyses as reported by the central laboratory, which were available from urine samples collected within 2 days before biopsy in 9 of 10 cases, are given in eTable 2 in the Supplement. Notably, in none of these cases were any findings other than isomorphic RBCs, WBCs, squamous cells, bacteria, and hyaline casts reported by the laboratory.

**Table 2.** Disease Process Suspected by Nephrologists and Clinical Diagnosis Made After Kidney Biopsy^a^

Case	Sex	Age, decade	eGFR, mL/min/1.73 m^2^	Proteinuria, g/g creatinine	Suspected disease process	Clinicopathologic diagnosis
Patient 1	Male	50s	35	0.2	Acute glomerulonephritis (10 of 14)	Immune complex-mediated glomerulonephritis secondary to bacterial infection; acute tubular injury and acute interstitial inflammation also present
					Acute interstitial nephritis (2 of 14)	
					Acute tubular necrosis (1 of 14)	
					Nondiagnostic (1 of 14)	
Patient 2	Female	20s	84	3.5	Nephrotic syndrome (14 of 14)	Lupus membranous nephropathy
Patient 3	Female	50s	100	7.2	Nondiagnostic (8 of 14)	Diffuse and nodular diabetic glomerulosclerosis
					Nephrotic syndrome (4 of 14)	
					Nonnephrotic proteinuria (1 of 14)	
					Acute tubular necrosis (1 of 14)	
Patient 4	Male	40s	38	0.3	Urinary tract infection (11 of 14)	Severe chronic-active interstitial nephritis attributed to immune checkpoint inhibitor therapy. Acute tubular necrosis also seen
					Acute interstitial nephritis (1 of 14)	
					BK nephropathy (1 of 14)	
					Nondiagnostic (1 of 14)	
Patient 5	Male	60s	95	2.2	Nephrotic syndrome (6 of 14)	Chronic-active thrombotic microangiopathy. Acute tubular injury also noted
					Acute tubular necrosis (3 of 14)	
					Nondiagnostic (2 of 14)	
					Acute interstitial nephritis (1 of 14)	
					Acute glomerulonephritis (1 of 14)	
					Drug-induced crystal nephropathy (1 of 14)	
Patient 6	Female	20s	126	1.6	Acute tubular necrosis (6 of 14)	Mesangial proliferative and membranous lupus nephritis
					Nondiagnostic (3 of 14)	
					Nephrotic syndrome (2 of 14)	
					Urinary tract infection (2 of 14)	
					Acute glomerulonephritis (1 of 14)	
Patient 7	Female	70s	39	0.1	Acute glomerulonephritis (9 of 14)	Antineutrophil cytoplasmic antibody-associated glomerulonephritis; moderate associated acute interstitial inflammation
					Acute interstitial nephritis (3 of 14)	
					Urinary tract infection (1 of 14)	
					Non-diagnostic (1 of 14)	
Patient 8	Male	40s	108	Unavailable	Nephrotic syndrome (14 of 14)	Primary membranous nephropathy
Patient 9	Female	50s	48	0.4	Nondiagnostic, bland, prerenal (12 of 14)	Mild features of diabetic nephropathy, mild acute tubular injury
					Acute tubular necrosis (2 of 14)	
Patient 10	Male	50s	65	0.5	Acute glomerulonephritis (14 of 14)	Thin basement membrane disease and mild IgA nephropathy without active inflammation

Abbreviation: eGFR, estimated glomerular filtration rate.

^a^ Baseline characteristics of patients, which were not revealed to the nephrologists, are also shown.

One reviewer reported examining the urine sediment 1 to 2 times per month, 3 estimated doing so 3 to 4 times per month, and 10 reported doing so 5 or more times per month. All believed that their manual examination of the urine sediment provided them with useful clinical information beyond what could be obtained from examining the urine microscopy report from their hospitals’ laboratories. All were confident in their ability to interpret urine sediment findings (eTable 1 in the Supplement).

## Discussion

Our study suggests that interobserver reliability of different urine sediment findings varies widely. Agreement ranged from slight for mixed cellular casts to almost perfect for squamous epithelial cells. For most sediment findings, moderate or substantial agreement was observed, as demonstrated by the overall κ estimates for casts and other sediment particles. Notable exceptions, however, included several sediment findings traditionally regarded as being of high clinical relevance during evaluation of patients with kidney disease, including WBC casts, RBC casts, and kidney tubular epithelial cells, in which fair agreement was observed. Though the kidney biopsy represents the reference standard diagnostic test for intrinsic kidney disease, agreement among pathologists on individual histopathologic lesions and diagnoses has been reported to range widely, with relatively low κ coefficients, for example, of 0.07 to 0.57 in a study of kidney procurement biopsies during deceased donor kidney transplantation and of 0.35 for diagnosis of acute interstitial nephritis.^9,10,11,12,13^

Adequate interobserver reliability of individual types of urine sediment particles is a prerequisite for the particles to serve as useful biomarkers. If interobserver reliability is poor, test performance characteristics may be adversely affected and inconsistent between examiners. Given the limited available data on diagnostic test performance characteristics of many urine sediment elements, suboptimal interobserver reliability could also raise questions about the generalizability of published findings in this field unless validated by more than 1 group of researchers. As an example, studies have shown how a urine sediment score based on a count of granular casts and kidney tubular epithelial cells can help discriminate prerenal injury from acute tubular necrosis among hospitalized patients with acute kidney injury and, moreover, predict its severity.^14,15^ Although these studies report how granular casts and kidney tubular epithelial cells can have utility as biomarkers in a common clinical scenario, and related studies of others have shown consistent findings,^16,17^ the modest interrater reliability for identifying kidney tubular epithelial cells in our study might suggest that a score relying partially on their correct count can be difficult to accurately assign, at least in a consistent manner among different nephrologists. Further standardization and education about the interpretation of epithelial cells in urine sediment may be of clinical value.

In 2009, Wald et al^6^ found that the interobserver reliability of urine sediment interpretation varied substantially between different types of findings. Overall, however, they reported lower κ statistics than we found in our study. For example, Wald et al^6^ reported κ statistics of 0.29 for isomorphic RBCs, 0.52 for hyaline casts, and 0.22 for course granular casts. There are several possible reasons for the differences between findings of Wald et al^6^ and ours, including higher image quality in our study and our use of short video clips and occasional polarized images. We also recruited many expert nephrologists known for their interest in urine sediment examination and teaching. Notably, studies by Secchiero et al,^18^ and Fogazzi et al^19,20^ from Italy, in which images of urine sediment particles were interpreted by laboratory personnel, reported variable but often excellent percent agreement. In Secchiero et al,^18^ the percentages of reviewers who correctly identified isomorphic RBCs, hyaline casts, and granular casts were 84.7%, 89.5% and 74.9%, respectively.

Our report of the concordance between nephrologists’ identification of the underlying disease process and the biopsy results should be understood as exploratory given the small number and selected nature of urine samples. We chose urine samples that had an abundant number of casts or cells rather than consecutive samples, and did not offer any clinical context which would likely have artificially improved the apparent concordance. Nevertheless, imaging the urine sediment of patients undergoing biopsy offered the opportunity to compare what nephrologists suspected to be the most likely disease process based solely on their review of the urinalysis to biopsy results. Although responses were typically varied, they matched well with biopsy results in several instances, particularly in cases of proliferative or nonproliferative glomerulonephritis. Our selection of cases, however, was primarily of glomerular diseases because we selected patients undergoing clinically indicated biopsy, and the nephrologists’ knowledge of common indications for kidney biopsy may have been a factor in their responses.

Over the past decades, manual examination of the urine sediment by clinicians has to a large extent been superseded by automated analysis. Lack of time and access to appropriate equipment may be factors in this development, particularly in private practice. Other possible reasons for this gradual change include regulations in the US such as the Clinical Laboratory Improvement Act and that the urine sediment examination may not be a billable procedure.^21^ This change in practice has raised concerns about potentially reduced competency among the physician workforce in performing this test.^3,4,22^ Most urine samples are now processed in central laboratories with workflows built around automation in which additional manual review is performed by laboratory technicians as needed, primarily if samples are flagged for unusual findings by the analyzers. While commonly used automated analyzers reliably detect and count certain elements of the urine, including WBCs, RBCs, bacteria, and squamous epithelial cells, they are known not to reliably detect many others, including dysmorphic RBCs, cellular casts, and crystals.^23,24,25,26,27^ This finding was borne out in our study as urinalysis reports from our hospital’s central laboratory did not indicate many abnormal results identified by the nephrologists. A study by Tsai et al^28^ found that urinalyses performed by 2 nephrologists blinded to clinical information were superior to laboratory-based urinalyses performed manually by technicians. Our results suggest that the sediment examination of practicing nephrologists still outperforms that performed by automated analyzers at hospital laboratories. Further studies of the diagnostic yield of manual vs automated urinalyses during workup of patients with kidney disease are needed. The incremental value of the urine sediment examination when added to other clinical information also requires further investigation, which could ideally be studied in a prospective multicenter study examining whether the addition of manual urine microscopy to other clinical data alters treatment decisions and improves diagnostic accuracy and patient outcomes.

### Limitations

This study type has limitations. Percent agreement, while readily interpreted, does not account for agreement by chance and tends to be inflated. The Fleiss κ, which provides a measure of agreement among multiple raters beyond that expected by chance, comes with a different set of limitations. It can be affected by uneven prevalence of the features being categorized and produce unrepresentatively low κ statistics for findings that are much less often chosen than the rest.^29^ This effect can be observed to an extent in our data, for example for mixed cellular casts and bacteria, which were less frequently depicted in the surveys. Hence, we believe that it is informative to view percent agreement and the total frequency of different responses alongside Fleiss κ. The more uneven distribution of different sediment findings in the study by Wald et al^6^ may also be a factor in the lower calculated κ statistics in their study.

This study has additional limitations to the intrinsic limitations of statistics for measuring interobserver reliability. We asked for a single response to casts, which could conceivably contain features of 2 distinct casts (eg, a hyaline cast containing rare lipid droplets). When such borderline casts are forcibly categorized, their calculated reliability may appear lower than if no potential overlap existed. Many of the nephrologists who participated in our study had known expertise in this field and may not be representative of most nephrologists in the US. We would likely have found less interobserver agreement with a less expert group of participants. Even though we used a high-resolution camera and included videos and still images for a detailed and realistic view, the examination of images and videos on a computer screen does not fully mimic the direct examination of the sediment under a microscope. Our study measured interobserver reliability but not the ability of nephrologists to find sometimes rare particles on a microscopy slide. Last, bright-field microscopy as used in our study does not provide the same level of detail as phase-contrast microscopy, but as bright-field microscopy may be more commonly available to clinicians our results may reflect the reality of current practice.

## Conclusions

In this diagnostic study, the interobserver reliability of different urine sediment findings among nephrologists was mostly moderate to substantial but varied substantially. For some findings, such as WBC casts and kidney tubular epithelial cells, only fair agreement was observed. Methods to improve interobserver reliability, which could involve established techniques such as phase-contrast microscopy or novel approaches such as artificial-intelligence assisted image analysis, should be pursued and appropriately studied. The diagnostic utility of the manual urine sediment examination by nephrologists should be further examined and compared with that of laboratory-based automated analyzers. The importance of maintaining competency among clinicians in performing this time-honored test can then be objectively assessed.

## Notes

Supplement. **eTable 1.** Nephrologist Reviewers’ Views on the Manual Urine Sediment Examination, and Their Current Use of It in Clinical Practice **eTable 2.** Microscopy Results as Reported by the Hospital’s Central Laboratory for Each of the 10 Cases Included
