# Supplementary material for: Assessment of Interobserver Reliability of Nephrologist Examination of Urine Sediment
Source: JAMA Netw Open. 2020 Aug 21;3(8):e2013959. doi: 10.1001/jamanetworkopen.2020.13959 (PMC7442930; doi:10.1001/jamanetworkopen.2020.13959)
Supplement: Supplement. — eTable 1. Nephrologist Reviewers’ Views on the Manual Urine Sediment Examination, and Their Current Use of It in Clinical Practice eTable 2. Microscopy Results as Reported by the Hospital’s Central Laboratory for Each of the 10 Cases Included [file jamanetwopen-3-e2013959-s001.pdf]

Supplementary Online Content

Palsson R, Colona MR, Hoenig MP, et al. Assessment of interobserver reliability of nephrologist examination of urine sediment. *JAMA Netw Open*. 2020;3(8):e2013959. doi:10.1001/jamanetworkopen.2020.13959

eTable 1. Nephrologist Reviewers' Views on the Manual Urine Sediment Examination, and Their Current Use of It in Clinical Practice

eTable 2. Microscopy Results as Reported by the Hospital's Central Laboratory for Each of the 10 Cases Included

This supplementary material has been provided by the authors to give readers additional information about their work.

Supplementary Material

eTable 1. Nephrologist reviewers' views on the manual urine sediment examination, and their current use of it in clinical practice.

**Question**	**Responses (% of N=14)**
Is equipment for examination of the urine sediment available to you when you are on inpatient service?	Yes: 100%
Is equipment for examination of the urine sediment available to you at your outpatient clinic?	Yes: 93%
On average, how many times per month do you perform manual urine sediment examinations?	"Almost never": 0%<br>"1–2 times per month": 7%<br>"3–4 times per month": 21%<br>"5 or more times per month": 71%
When you consult on a new patient in the hospital with acute kidney injury, how often do you estimate you perform a manual examination of the urine sediment as part of your workup?	≤25% of consults: 14%<br>>25 but ≤50% of consults: 7%<br>>50 but ≤75% of consults: 21%<br>>75% of consults: 57%
When you consult on a new patient with chronic kidney disease at your outpatient clinic, how often do you estimate you perform a manual examination of the urine sediment as part of your workup?	≤25% of consults: 21%<br>>25 but ≤50% of consults: 36%<br>>50 but ≤75% of consults: 14%<br>>75% of consults: 29%
What is your view of the following statement: "My manual examination of the urine sediment provides me with useful information during workup of patients with kidney disease, beyond what I obtain from looking at the urine microscopy report from my hospital's laboratory"	"Strongly disagree," "Disagree," or "Neutral": 0%<br>"Agree": 36%<br>"Strongly agree": 64%
How confident do you feel overall in your ability to interpret urine sediment findings on the scale of 1-5 (1 being "not at all confident" and 5 being "highly confident")	1, 2, or 3: 0%<br>4: 64%<br>5: 36%

© 2020 Palsson R et al. *JAMA Network Open.*

eTable 2. Microscopy results as reported by the hospital's central laboratory for each of the 10 cases included. All shown results are from urine samples processed by the laboratory within 2 days before biopsy. *RBC, red blood cell; WBC, white blood cell; LPF, low-power field; HPF, high-power field.*

**Case**	**1**	**2**	**3**	**4**	**5**	**6**	**7**	**8**	**9**	**10**
**Microscopy results as reported by the hospital's central lab**	*RBC* 26/HPF<br><br>*WBC* 14/HPF<br><br>*Bacteria* Trace<br><br>*Squamous cells* Trace<br><br>*Hyaline casts* 7/LPF	*RBC* 1/HPF<br><br>*WBC* 3/HPF<br><br>*Bacteria* Trace<br><br>*Squamous cells* 2+<br><br>*Hyaline casts* 4/LPF	*RBC* 1/HPF<br><br>*WBC* 0-2/HPF<br><br>*Bacteria* Trace<br><br>*Squamous cells* Trace<br><br>*Hyaline casts* 0–2/LPF	*RBC* 4/HPF<br><br>*WBC* 26/HPF<br><br>*Bacteria* Trace<br><br>*Squamous cells* Negative<br><br>*Hyaline casts* 0–2/LPF	*RBC* 1/HPF<br><br>*WBC* 1/HPF<br><br>*Bacteria* Negative<br><br>*Squamous cells* Trace<br><br>*Hyaline casts* 1/LPF	*RBC* 1/HPF<br><br>*WBC* 1/HPF<br><br>*Bacteria* Trace<br><br>*Squamous cells* Trace<br><br>*Hyaline casts* 0–2/LPF	*RBC* 1/HPF<br><br>*WBC* 0-2/HPF<br><br>*Bacteria* Trace<br><br>*Squamous cells* Negative<br><br>*Hyaline casts* 0–2/LPF	*RBC* 8/HPF<br><br>*WBC* 1/HPF<br><br>*Bacteria* Trace<br><br>*Squamous cells* Negative<br><br>*Hyaline casts* 0–2/LPF	Not available close to the time of biopsy	*RBC* 30/HPF<br><br>*WBC* 1/HPF<br><br>*Bacteria* Negative<br><br>*Squamous cells* Negative<br><br>*Hyaline casts* 0–2/LPF

A **survey example** can be found online at https://forms.gle/WZKPzHF7nPd5uN897.

© 2020 Palsson R et al. *JAMA Network Open.*
